# Evaluation of sleep disorder and its effect on sexual dysfunction in patients with Fibromyalgia syndrome

**DOI:** 10.4274/tjod.17047

**Published:** 2016-12-15

**Authors:** Tuba Tülay Koca, Günseli Karaca Acet, Emrullah Tanrıkut, Burcu Talu

**Affiliations:** 1 Malatya State Hospital, Clinic of Physical Medicine and Rehabilitation, Malatya, Turkey; 2 Malatya State Hospital, Clinic of Obstetrics and Gynecology, Malatya, Turkey; 3 İnönü University Faculty of Health Sciences, Department of Physical Medicine and Rehabilitation, Malatya, Turkey

**Keywords:** Fibromyalgia syndrome, Sexual dysfunction, sleep disorder

## Abstract

**Objective:**

Sexual problems are commonly seen in women with fibromyalgia syndrome (FMS). The objective of this study was to reveal the relationship between the severity of symptoms, sleep disorder, and sexual dysfunction in women with FMS.

**Materials and Methods:**

A total of 140 sexually active women with FMS aged 17-67 years who presented to our physical medicine and rehabilitation outpatient clinic between January 2016 and June 2016 were enrolled in the study. The patients’ age, height, body weight, body mass index (BMI), and general pain score [visual analogue scale, (VAS)] for the last 1 week were recorded. The patients were given three different sets of questionnaires: the Pittsburgh Sleep Quality Index (PSQI), Fibromyalgia Impact Questionnaire (FIQ), and Female Sexual Function Index (FSFI).

**Results:**

The mean age of the patients was 40.3±8.5 years; the mean BMI was 27.1±4.4 kg/m^2^, VAS (last 1 week) was 6.9±2 cm, the mean PSQI was 24.8±10.8 (one patient with PSQI ≤5), FIQ was 65.9±19.2, and FSFI was 19.0±6.9. No significant relationship was observed between the mean PSQI and BMI values (p=0.401), whereas a significant relationship was found between the mean values of VAS, FIQ, and FSFI (p=0.03; p=0.034; p<0.001, respectively). In Pearson’s correlation analysis, a positive correlation was noted between PSQI and VAS (r=0.324; p<0.001) and FIQ values (r=0.271; p=0.001). A significant relationship was found between the FIQ and VAS values (p<0.001). P less than 0.005 was considered statistically significant.

**Conclusion:**

Sleep disorder is regarded as the underlying cause for many signs and symptoms in FMS. Sexual dysfunction may develop in women with FMS, based on the severity of the disease and poor sleep quality. We found that sleep dysfunction was significantly related with the severity of disease, pain, and sexual disfunction. We also found a positive correlation between VAS and PSQI.

## INTRODUCTION

Fibromyalgia syndrome (FMS) is an entity with multiple concomitant disorders, rather than a single disorder. The common symptoms of FMS include sleep disorders, affective disorders, chronic generalized pain, and fatigue. The pathophysiology of FMS has yet to be elucidated, and no treatment is available for relieving all of the symptoms^(1)^. Several studies in the literature have found a close correlation between FMS and sexual dysfunction. The main symptoms include decreased sexual drive, excitement, orgasm, and increased genital pain^(2)^. From a psychological point of view, stress, chronic generalized pain, fatigue, and sleep disorder negatively affect the sexual life of patients with FMS. In addition, the medicines used for treating the disease are also known to negatively affect sexual function^(2)^. Sleep problems are one of the most common symptoms in patients with FMS^(3)^. This study reviews the relationship between disease progress, sleep problems, and sexual dysfunction.

## MATERIALS AND METHODS

A total of 140 sexually active, pre- or postmenopausal women who presented to our physical medicine and rehabilitation outpatient clinic between January 2016 and June 2016 who was diagnosed FMS in accordance with the American College of Rheumatology 1990^(4)^ and 2010 criteria, were enrolled in the study. The exclusion criteria included pregnancy, breastfeeding, major depression, active infection or inflammation, and malignancies. The patients’ age, height, body weight, body mass index (BMI), and general pain score for the last 1 week were recorded. The patients were given three different sets of questionnaires: the Pittsburgh Sleep Quality Index (PSQI), Fibromyalgia Impact Questionnaire (FIQ), and Female Sexual Function Index (FSFI) in order to evaluate sleep function, disease severity, and sexual function.

The FIQ was developed by Burckhardt to evaluate the functional status, disease progression, and outcomes of patients with fibromyalgia. The Turkish version of the FIQ was validited by Sarmer et al.^(5)^. The scale is used to follow up the conditions and outcomes of patients with FMS. The first item consists of 10 Likert-type questions. In the second and third items, the patient is asked to tick days to allow for the determination of “disease exposure” and “absence from work.” The scores obtained are adapted to 10. The remaining seven questions are based on marking the corresponding points in the equivalent visual scale. The scoring interval is 0-100.

The PSQI scale provides information about the type and severity of sleep disorder and sleep quality in the last 1 month. Out of a total of 24 questions, 19 questions are answered by the patient, and the remaining 5 are answered by the partner of the patient. Using the 19 questions answered by the patient, 7 subdimensions are evaluated, including the subjective sleep quality, sleep latency, sleep duration, routine sleep activity, sleep disorder, use of sleeping pills, and daytime dysfunction. Each item in the scale is graded from 0 (no problem at all) to 3 (severe problem). The total scores for the seven subdimensions give the total PSQI score. A total score of 5 and less indicates that the sleep quality is “good”^(6)^. The Turkish validity of the scale was provided by Agargun et al.^(7)^. The FSFI was developed by Rosen et al.^(8)^ in 2000 as a multidimensional scale consisting of 6 parts and 19 items to assess sexual function in women. Six dimensions are involved in the scale: desire, excitement, lubrication, orgasm, satisfaction, and pain. The lowest score on the scale is 2.0, and the highest is 36.0. The coefficient is 0.6 for the first and second questions; 0.4 for questions 3-10; and 0.3 for questions 11-19. The study was approved by the local Ethics Committee (2015-58) and was performed in accordance with the ethics standards described in an appropriate version of the 1975 Declaration of Helsinki. 

### Statistical Analysis

SPSS version 21 (IBM Corp. Released 2012. IBM SPSS Statistics for Windows, Armonk, NY: IBM Corp.) was used for data analysis. The normal distribution of data was checked using the Kolmogorov-Smirnov test. A comparison was made for each parameter. The t test was used for normally distributed groups, and the Mann-Whitney U test was used for abnormal distribution. Chi-square analyses were used for a detailed examination of differences across groups. We used the one-way ANOVA test to compare continuous variables. Regression analyses were run for comparing multiple variables. Pearson’s correlation analysis was used as the correlation analysis. The significance of these tests was defined as p≤0.05.

## RESULTS

A total of 140 sexually active women aged 17-67 years who were diagnosed as having FMS were enrolled in this study. The mean age of the patients was 40.3±8.5 years, the mean BMI was 27.1±4.4, the general pain score [visual analogue scale, (VAS)] in the last 1 week was 6.9±2 cm, the mean PSQI was 24.8±10.8 (one patient with PSQI ≤5), the mean FIQ was 65.9±19.2, and the mean FSFI was 19.0±6.9. The descriptive data of the study are summarized in Table 1. Based on the results of the Kolmogorov-Smirnov test, it was observed that the FIQ, FSFI, and PSQI data did not have a normal distribution (p=0.032; p<0.001; p=0.002, respectively) (Table 2). All parameters were compared with each other using regression analysis and the Mann-Whitney U test. No significant relationship was observed between the mean PSQI and BMI values (p=0.401), whereas a significant relationship was found between the mean VAS values (last 1 week), FIQ, and FSFI (p=0.03; p=0.034; p<0.001; respectively) (Table 3).

In the Pearson’s correlation analysis, a positive correlation was found between the PSQI and VAS (r=0.324; p=<0.001; p<0.001) and FIQ values (r=0.271; p=0.001). No correlation was found between the PSQI and FSFI values (p=0.645). The FSFI values were compared with all other parameters using regression analysis and the Mann-Whitney U test. No statistically significant relationship was observed between the FSFI, FIQ, and BMI values (p=0.183; p=0.682, respectively) (Table 3). A significant relationship was observed between the FSFI and PSQI values, age (in favor of younger ages) (p<0.001); no correlation was determined (r=0.27, p=0.325; r=0.57, p=0.251). A significant relationship was found between FIQ and VAS values (last 1 week) (p<0.001) (Table 3). A p value <0.005 was considered statistically significant.

## DISCUSSION

Many functions of sex hormones may affect chronic pain syndromes such as migraine, headaches, FMS, inflammatory arthritis and back ache, and others^(8)^. Scientific research has shown sex-specific differences in pain sensitivity and threshold. Although the underlying pathogenetic mechanism responsible for these differences has not yet been defined, the probability of a sex hormone effect on the nociceptive process has attracted attention^(9)^.

The pathophysiology of FMS includes alterations in ascending and descending central nervous and peripheral nerve pathways, which lead to increased pain and sensitivity. Research on the risk factors has focused on several genetic predispositions and the effects of stress and poor sleep^(10)^. Recent human neuroimaging studies suggested that FMS, a chronic widespread pain disorder, exhibited altered thalamic (modulation of pain) structure and function^(11)^. The exact etiology of FMS is unknown, and the pathogenesis involves psychology, environmental, genetics, hormonal (serotonin), and impaired sleep quality. Brooks et al.^(12)^ found that gynecologic, endocrine, and autoimmune diagnoses were associated with a diagnosis of FMS. They also found a relationship between the timing of gynecologic surgery and pain onset in FMS. Patients with FMS commonly have various autoimmune, endocrine, gynecologic, or psychiatric disorders. Sexual dysfunction related to chronic fatigue syndrome has become an increased concern rceently; however, limited studies have analyzed this subject to date. FMS may cause sexual dysfunction as a result of impaired emotional state. Whether the partners of female patients with FMS also have impaired sexual life has been the subject of various investigations^(13)^. Blazquez et al.^(14)^ analyzed the sexual function of 615 patients with chronic fatigue syndrome, and remarked that frequency of sexual dysfunction with FMS, Sjögren syndrome, and Myofacial pain syndrome concerned cognitive, neurologic, and neurovegetative symptoms were higher.

Chronic generalized pain is the cardinal symptom of FMS, and results in decreased quality of life, along with physical and psychosocial impairments. Burri et al.^(15)^. reported difficulty in lubrication, sexual pain, and increased sexual stress in female patients with FMS who presented with chronic generalized pain. Terzi et al.^(16)^ determined a lower threshold of pain and a higher number of tender points in women with FMS who presented with dyspareunia compared with those without dyspareunia. The central pathophysiology in the development of dyspareunia in female patients with FMS still needs further investigation. Ghizzani et al.’s^(17)^ results supported that coital pain develops with the severity of FMS symptoms depending on the cooperative effect of peripheral and central sensitization mechanisms in female patients with FMS.

Palagini et al.^(1)^ hypothesized that sleep disorders activate stress and inflammation systems, playing a central role in all other symptoms. This also accounts for the high frequency of togetherness with pain, sleep, and mental disorders. Starting from this point of view, it is suggested that the treatment of sleep disorders may help alleviate symptoms of FMS and mental disorders. In a study similar to the present study, which was performed by Amasyali et al.^(18)^ in 54 patients, a positive correlation was observed between the patients with PSQI score >5 (poor sleep quality) and sexual dysfunction. Additionally, a marked link was found between the high FIQ scores and sexual dysfunction. The present study found poor sleep quality to be markedly associated with the pain score, severity of disease, and sexual dysfunction. No marked link was found between BMI and sleep disorder and sexual dysfunction. The rates of sexual dysfunction and poor sleep quality are higher at older ages. Hence, it is concluded that reductions in generalized pain, severity of disease, and sleep disorder may also reduce sexual problems in patients. In FMS, multiple symptoms are closely associated with each other, and sleep disorder appears to play a main function in the development of all other symptoms. FMS is a chronic pain syndrome characterized by subjective primary insomnia. It shows abnormalities in the continuity and architecture of sleep in polysomnographic findings. Reduced quality of sleep, increased awakening, reduced slow-wave sleep, and emergence of abnormal alpha waves (alpha-delta) in nonrapid eye movement sleep are seen in sleep recordings^(19)^. FMS symptoms are considered to be related to nonrestorative sleep disorder associated with alpha-electroencephalogram sleep disorders. Additionally, patients with FMS may also report sleep disorders such as sleep apnea or periodic limb movements^(19,20)^. Diaz-Piedra et al.^(3)^ observed a lower sleep quality, a greater wake phase, and a higher number of awakenings in a one-night polysomnographic evaluation of patients with FMS compared with a control group. It was also seen that patients reported poor subjective sleep quality^(3)^. Stuifbergen et al.^(20)^ evaluated the sleep disorders of 104 female patients with FMS and reported subjective sleep disorder in 44% of the patients and objective sleep disorder in 21%. In patients with objective sleep disorders, the pain score, tender point index, and FIQ scores were higher, and also more depressive symptoms were observed compared with the others.

Miro et al.^(21)^ studied whether patients with FMS had different cognitive alterations depending on their sex. According to the study, treatments aimed at decreasing emotional distress seemed to improve attention more in women than in men; those intended to improve sleep quality were likely to reduce alertness incompetency in women and executive problems in men. Similar to the literature, we found a statistically significant association with sleep disfunction, disease severity, pain, and sexual disfunction. In contrast to the literature, we found a positive correlation between sleep disfunction, disease severity, and pain. It has been estimated that pain is positively correlated with disease severity and poor sleep quality. According toour findings, BMI has no effect on sleep, disease severity, pain and sexual function. Our patient population was overweight and middle-aged to fit with FMS. We also found fewer sexual disfunction symptoms at younger ages.

The treatment of FMS involves pharmacologic (tricyclic antidepressants, antiepileptic agents, selective serotonin uptake inhibitors) and nonpharmacologic (massage, exercise, acupuncture) therapies^(10)^. No drug is recuperative for all of the symptoms of FMS. When scheduling the treatment plan, it is necessary to take into consideration that FMS consists of multiple closely-associated symptoms^(10)^. Therefore, behavioral programs should be included to increase (deep sleep) and improve poor sleep quality, which is the most common symptom. Therapeutic measures should be taken, the adverse effects of drugs should be minimized, and a multidisciplinary approach should be used to improve the patient’s quality of life.

### Study Limitations

First, our patients’ age range was very wide (17 years up to 67 years old). Age has a major affect on both sleep quality and sexual life. The study includes both pre- and post-menopausal women. The status of menopause is another confounding factor that is in itself a cause for worse sexual domain scores compared with premenopausal women. In addition, related to menopause, some other sexual function questionnaires were developed for menopausal women. We did not consider the menopausal status of our patients with any questionnaire or laboratory examinations. Additionally, medication may have affected our study results; we did not ask about medicine use for menapause or FMS. The study is a single-centered study and our clinic is located at one of the eastern cities in Turkey. Literate patients filled out scales by themselves, but most illiterate patients did not want to complete the FSFI scale; therefore, we had to exclude them. It would be have been better if we had asked about the patients’ educational levels.

## CONCLUSION

Sleep disorder is regarded as the underlying cause for many signs and symptoms in FMS. Sexual dysfunction may develop in women with FMS due to the potency of the disease and poor sleep quality. We found that sleep dysfunction was significantly related with the severity of disease, pain and sexual disfunction. We also found a positive correlation between VAS and PSQI (when pain scor is higher, sleep disorder is seen more prevelant). Therefore, beyond others, the treatment of sleep disorder is of vital importance in the management of FMS.

## Figures and Tables

**Table 1:**
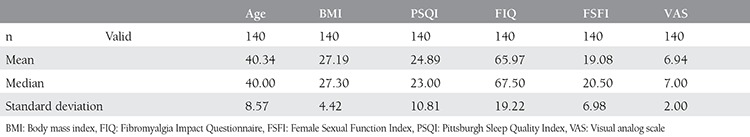
Descriptive data

**Table 2:**
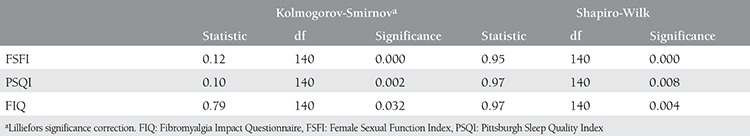
Tests of normality

**Table 3:**
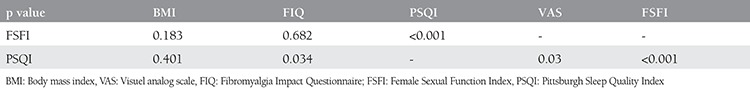
Multiple Comparisons of the parameters
